# Molecular Crowding Stabilizes DNA Coacervate Droplets and Generates Self-Organized Characteristic Patterns

**DOI:** 10.1021/jacsau.6c00746

**Published:** 2026-07-27

**Authors:** Naoki C. Yoshida, Mayu Shono, Kenichi Yoshikawa, Masahiro Takinoue

**Affiliations:** † Department of Life Science and Technology, 13290 Institute of Science Tokyo, 4259 Nagatsuta-cho, Midori-ku, Yokohama, Kanagawa 226-8501, Japan; ‡ Komaba Institute for Science, Graduate School of Arts and Sciences, 13143 The University of Tokyo, Komaba 3-8-1, Meguro, Tokyo 153-8902, Japan; § Faculty of Life and Medical Sciences, Doshisha University, Kyoto 610-0394, Japan; ∥ Laboratory for Chemistry and Life Science, Institute of Integrated Research, 13290 Institute of Science Tokyo, 4259 Nagatsuta-cho, Midori-ku, Yokohama, Kanagawa 226-8501, Japan; ⊥ Research Center for Autonomous Systems Materialogy (ASMat), Institute of Integrated Research, 13290 Institute of Science Tokyo, 4259 Nagatsuta-cho, Midori-ku, Yokohama, Kanagawa 226-8501, Japan

**Keywords:** Bottom-up synthetic biology, Liquid−liquid phase separation, Biomolecular coacervates, Molecular crowding, DNA coacervates, DNA droplets, DNA condensates

## Abstract

Living cells are characterized by highly crowded intracellular environments that are rich in macromolecules. Liquid-like biomolecular condensates play a central role in regulating biochemical reactions in such environments. However, the effects of macromolecular crowding on condensate formation and properties remain inadequately understood, despite their importance for both the fundamental understanding of biomolecular condensates and the development of artificial cells and micromachines. In this study, we systematically investigated the influence of polymeric crowding agents—polyethylene glycol (PEG), dextran, and Ficoll—on liquid-like DNA condensates (DNA droplets) formed through the sticky-end hybridization of Y-shaped DNA nanostars. We show that PEG, above the molecular weight of several kilograms (k), significantly enhances the thermal stability of DNA droplets compared to dextran and Ficoll, whereas low-molecular-weight PEG (0.6k and 1k) tends to destabilize droplet formation. In contrast, PEG 7.5k robustly promoted droplet formation and enhanced thermal stability. Furthermore, under PEG 7.5k crowding conditions, multiple types of DNA droplets composed of noncomplementary nanostars spontaneously assemble into adjacent and alternating network patterns. The resulting spatial organization is tunable by the PEG concentration, salt concentration, nanostar concentration, and number of droplet species. These findings reveal macromolecular crowding as an effective design parameter for controlling programmable DNA droplets, providing a versatile strategy for constructing functional microstructured systems relevant to micromachines and synthetic-cellular platforms.

## Graphical abstract

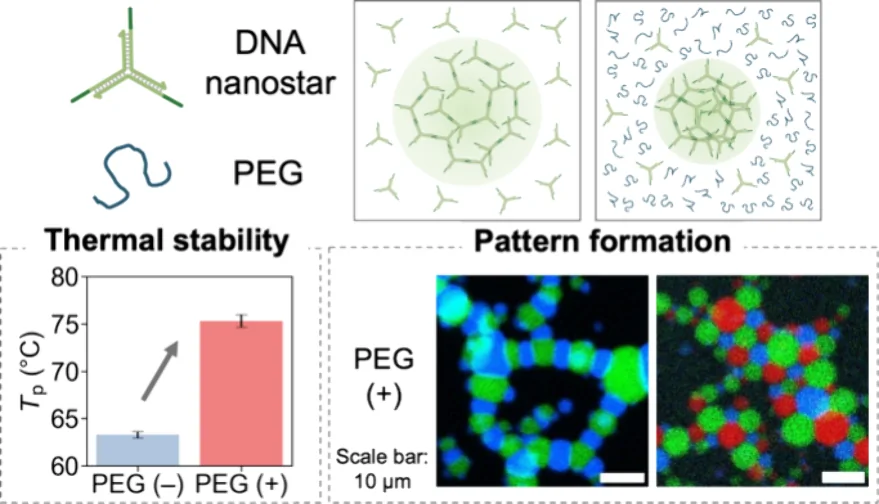

## Introduction

In recent years, liquid-like biomolecular condensates, also known as biomolecular droplets, have attracted considerable attention, particularly in the field of synthetic biology.^1,2^ These condensates primarily form through liquid–liquid phase separation (LLPS) via associative and segregative biomolecular interactions, known as associative and segregative LLPS, respectively. Biomolecular condensates play a crucial role in regulating intracellular processes;^3,4^ for instance, they activate or inhibit reactions through biomolecular localization,^5,6^ respond to stimuli such as heat or pH stress,^7,8^ and form internal patterns (subcompartments) that facilitate the spatiotemporal control of reactions.^9^ Owing to these characteristics, biomolecular condensates hold promise for applications in artificial cells and molecular robots, as well as for understanding intracellular functions. However, the intracellular environment is densely populated with biomolecules; for instance, the total macromolecular concentration in the *E. coli* cytoplasm has been estimated to reach 300–400 mg/mL.^10^ The effects of crowding on the formation, physical properties, and functions of biomolecular condensates remain poorly understood.^11,12^ Therefore, a deeper understanding of the effects of crowding is essential for the bottom-up construction of synthetic biomolecular condensates.

The influence of macromolecular crowding on biomolecular condensates has primarily been investigated using *in vitro* LLPS model systems that utilize synthetic polymeric crowding agents, including polyethylene glycol (PEG), dextran (Dex), and Ficoll.^11,12^ These crowding agents are recognized for their ability to induce or enhance LLPS, as demonstrated by PEG-induced condensation of globular proteins,^13,14^ dextran-enhanced LLPS of disordered triblock proteins,^15^ and Ficoll-dependent reduction of the critical concentration necessary for LLPS of RNA-binding proteins.^16^ Although numerous aspects of these mechanisms remain unclear, three primary factors have been proposed.^11,12^ First, LLPS is induced by depletion forces^17^ arising from macromolecular crowding, which effectively promotes condensate formation. Second, the presence of a crowding agent can alter protein solubility, as represented by the aqueous two-phase system of PEG and Dex.^18^ These two mechanisms are categorized as segregative phase separation. Third, when a crowding agent interacts with a protein, phase separation may be promoted via co-condensation.^19^ Moreover, it has been shown that crowded environments not only influence the behavior of condensate formation but also impact biophysical properties, such as increased condensate density,^20^ reduced internal mobility,^19^ and the pattern formations.^21,22^ Conversely, research on crowding effects has predominantly focused on protein-based condensates, where the complexity of intermolecular interactions hinders independent mechanistic evaluation and control. Consequently, LLPS model systems with programmable and predictable interactions are required.

The structural programmability enabled by specific Watson–Crick base pairing, combined with the predictability based on DNA hybridization thermodynamics, has enabled the assembly of three-dimensional structures via DNA hybridization. In particular, branched DNA nanostructures^23,24^ have been engineered to form static, solid-like DNA assemblies, such as dendrimers,^25,26^ crystals,^27^ nano- and microgels,^28,29^ and bulk hydrogels.^30^ Subsequently, owing to the clarification of the phase separation behaviors of branched DNA nanostructures,^31^ named DNA nanostars, as valency-controllable colloids, branched DNA nanostructures have been adapted to create liquid-like DNA condensates formed through LLPS.^32–34^ Recently, DNA condensates have been used as controllable LLPS models of biomolecular condensates,^31,35,36^ and it has been demonstrated that biophysical properties, such as stability,^32,33,37,38^ fusion,^38,39^ growth rates,^40^ and pattern formation,^41,42^ can be tuned through the intentional design of DNA sequences. Moreover, when coupled with specific molecular reactions, DNA condensates have been applied to dissolution/reassembly,^43,44^ division,^37,45,46^ bubbling,^47^ light-induced drug release^48^ and directional transportation,^49^ fluidic deformation and motion,^50–52^ and molecular information-processing functions such as sensing/computing^44,53^ and communication.^54^ Nevertheless, these findings have primarily been observed under dilute solution conditions, leaving the effects of crowded environments, such as intracellular conditions, on DNA condensates unexplored. *In vivo,* the expression of RNA condensates composed of RNA nanostars has been demonstrated in bacteria,^55^ a crowded intracellular environment. In the case of RNA condensates, PEG induced contact between two distinct condensate types *in vitro* leads to network formation (pattern formation).^56^ It has also been reported that positioning of DNA in droplets under LLPS is markedly dependent on its higher order structural change.^57^ However, systematic investigations of the effects of factors, such as crowding agent concentrations and coexisting immiscible condensates, on DNA condensates have not yet been undertaken.

In this study, we investigated the impact of representative crowding agents—PEG, Dex, and Ficoll—on DNA condensates formed via sticky-end (SE) hybridization of Y-shaped branched DNA nanostructures (3-arm DNA nanostars (NSs));^28,29^ hereafter, the term DNA droplet is used because we focus on the liquid-like state of DNA condensates, which is indicated by droplet fusion and the high internal mobility of NSs observed near the phase-separation temperature.^37–39^ PEG is well-known for inducing DNA condensation/compaction in the presence of salts,^58,59^ and it has been documented that phase separation in the DNA–PEG system occurs under microscale confinement.^60^ In this study, we revealed that PEG significantly enhanced the thermal stability of DNA droplets compared to Dex and Ficoll. Although low-molecular-weight PEG 0.6k and 1k destabilized DNA droplets, PEG 7.5k improved thermal stability and facilitated DNA droplet formation. Moreover, in the presence of PEG 7.5k, self-assembled patterns emerged; specifically, different types of DNA droplets composed of noncomplementary NSs were arranged adjacently and alternately, resembling structures reported in RNA condensates.^56^ These patterns varied based on PEG, salt, and NS concentrations, as well as the number of droplet types. These findings introduce a novel strategy for controlling DNA droplets using crowding agents that can act cooperatively with sequence-dependent programming.

## Results and Discussion

### Effect of Crowding Agents on Thermal Stability of DNA Droplets

Figure 1a shows the phase separation of DNA droplets through the SE hybridization of the NSs. The NSs show the phase separation below the phase-separation temperature (*T*_p_), while they disperse above *T*_p_. To investigate the influence of molecular crowding on DNA droplets, the phase-separation temperature was determined based on the observation by the confocal laser-scanning microscopy (Olympus) using 5 μM 3-arm DNA nanostar in the presence of representative crowding agents at a concentration of 10% (w/v), specifically PEG 7.5k (MW: 7.5 kDa), dextran 200k (MW: 200 kDa), and Ficoll 400k (MW: 400 kDa). The phase-separation temperature based on microscopy is named *T*_p,m_. Notably, 10% (w/*v*) is comparable to or exceeds the overlap concentration (c*; Figure S1) of each crowding agent. The radius of gyration (*R*_g_) values for PEG 7.5k, dextran 200k, and Ficoll 400k are approximately 3.9,^61^ 15,^62^ and 11 ^63^ nm, respectively. The corresponding c* are approximately 5, 2.4, and 12% (w/v), respectively. In the absence of crowding agents, the DNA droplets composed of NS formed below 63 °C as the temperature decreased from 85 °C at a rate of −1 °C/min (Figure 1b). In contrast, in the presence of 10% (w/v) PEG 7.5k, Dextran 200k, and Ficoll 400k, DNA droplets formed at temperatures below 76, 65, and 67 °C, respectively (Figure 1b). The number of DNA droplets was recorded in microscope images as the temperature decreased (Figure 1c), and *T*_p,m_ was determined as the temperature at which the count reached 5 droplets.

**Figure 1:**
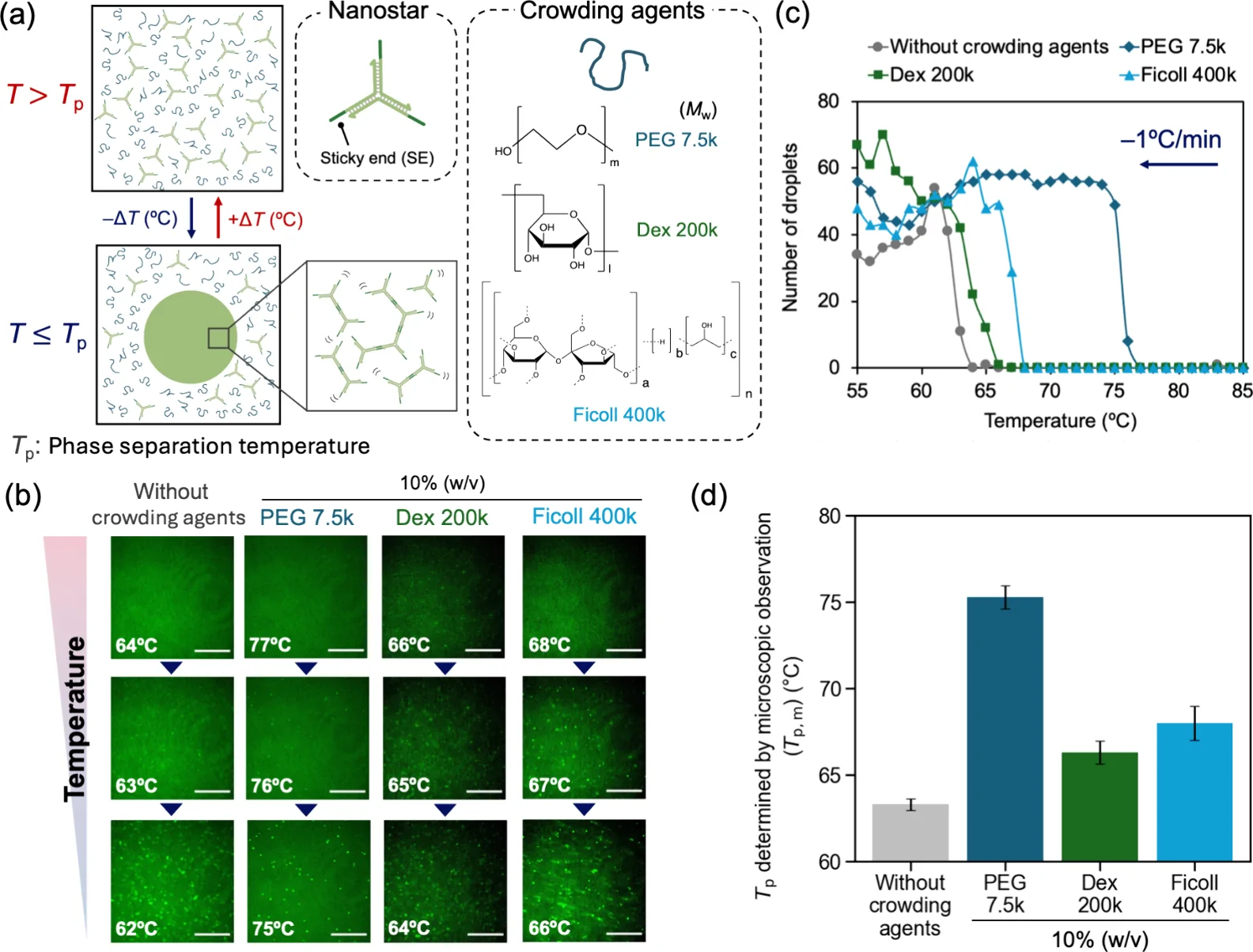
Effect of crowding agents on DNA droplet phase separation. (a) Temperature-dependent phase separation of Y-shaped branched DNA nanostructures (NSs). NSs form DNA droplets via sticky end (SE) hybridization. Proposed illustration of DNA droplets in a crowding-agent environment. Structural formulas of representative crowding agents, including polyethylene glycol (PEG), dextran (Dex), and Ficoll, are displayed. (b) Representative sequential images of DNA droplet formation as temperature decreased, shown (i) without crowding agents, and with 10% (w/v) of (ii) PEG 7.5k, (iii) Dex 200k, and (iv) Ficoll 400k. Scale bars: 40 μm. (c) Temperature-dependent number of DNA droplets obtained from (b). The number of droplets was defined as the count of droplets with a volume of ≥∼1.8 fL within an observation volume of approximately 34 pL. For example, when 34 droplets were counted, the droplet number density was 1/pL. The confocal laser-scanning microscope (Olympus) was used for observation. (d) The phase-separation temperature determined by microscopic observation (*T*_p,m_). Error bars represent the standard error (*n* = 3).

The observed increase in *T*_p,m_ in the presence of crowding agents (Figure 1d) is likely due to the condensation of NSs, which are induced by depletion effects. These effects arise from an entropic gain as the surrounding polymers regain translational freedom when NSs get together. In this context, PEG 7.5k significantly increased *T*_p,m_ compared to Dex 200k and Ficoll 400k (Figure 1d). This trend agrees with the fact that the depletion interactions are more pronounced for flexible polymers with higher configurational entropy, resulting in polymer-dependent variations in depletion efficiency.^64,65^ In addition, DNA has been reported to be less soluble in PEG than in Dex^66–68^ or Ficoll,^69,70^ suggesting enhanced PEG–DNA-driven segregative phase separation.

The phase-separation temperature based on turbidity (*T*_p,t_) was determined via the turbidity measurements (absorbance at 600 nm) using the UV–visible spectrophotometer. *T*_p,t_ is the temperature at which the turbidity began to increase during cooling from 99 to 25 °C at a rate of −1 °C/min. In the presence of 10% (w/v) PEG 7.5k, *T*_p,m_ and *T*_p,t_ were 75 and 73 °C, respectively, which were closely aligned (Figures 1c, 2a, and 2b). Additionally, the NS without SE did not form DNA condensates under microscopic observation (Figure S2a) and did not exhibit a transition point in the absorbance curve (Figure S2b). These results indicate that turbidity measurements yield similar results to microscopic observations and are effective for streamlining the measurement process of phase separation transition temperatures.

**Figure 2:**
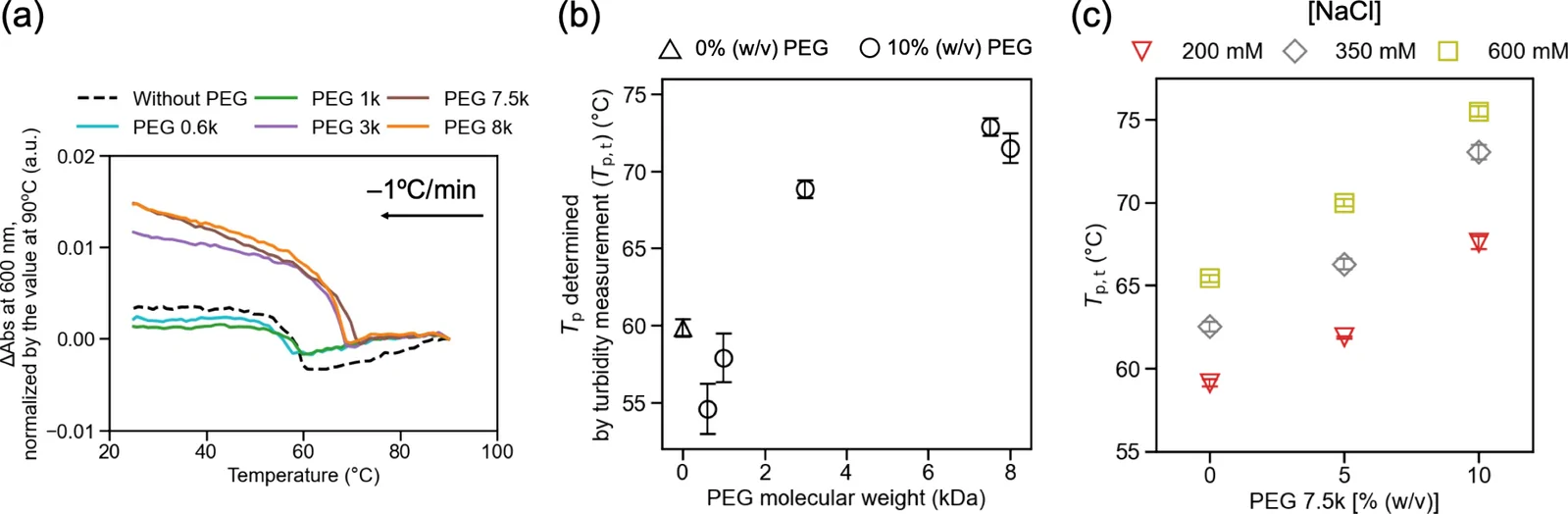
PEG molecular weight (MW)-dependent phase-separation temperature (*T*_p_) of DNA droplets. (a) Absorbance curves at 600 nm for NSs during cooling, both in the absence and presence of PEG having different MW. (b) *T*_p_ determined from the turbidity measurements in panel (a) (*T*_p,t_). (c) NaCl- and PEG 7.5 k-concentration-dependent *T*_p,t_ obtained from Figure S4. Error bars in (b) and (c) represent the standard error (*n* = 3).

To further examine the factors contributing to the stabilizing effect of PEG, the PEG concentration was maintained at 10% (w/v), and *T*_p,t_ was evaluated in relation to the MW of PEG. In the presence of PEG with MWs of 3–8k, *T*_p,t_ increased by approximately 10 °C compared to the conditions without PEG (Figures 2a and 2b). Conversely, PEG with MWs of 0.6k and 1k resulted in a decrease in *T*_p,t_ (Figures 2a and 2b). Consistently, DNA droplet formation was observed at the respective *T*_p,t_ for each PEG MW condition (Figure S3). A plausible explanation for the inability of low-molecular-weight PEGs (≤1k) to stabilize DNA droplets is that their small *R*_g_ results in a high overlap concentration (c*), such that c* is not reached even at 10% (w/v) (Figure S4a). Accordingly, *T*_p,t_ was evaluated at the overlap concentration for each PEG MW. Even under these conditions, the stabilizing effect of low-molecular-weight PEGs remained weak, and in some cases, a clear transition point could not be defined (Figures S4b and S4c). Consistent with this interpretation, previous studies have reported that low-molecular-weight PEGs (≤1k) reduce the thermal stability of 30-mer DNA duplexes,^71^ and this destabilization has been attributed to reduced water activity upon PEG addition. Based on these findings, PEG 7.5k, which effectively enhanced the thermal stability of DNA droplets, was predominantly used in this study.

Increasing the concentration of PEG 7.5k from 0 to 10% (w/v) consistently elevated *T*_p,t_ (Figures S5 and 2c). Notably, even under low-salt conditions—where DNA droplets formation is hindered by electrostatic repulsion between DNA strands—the addition of PEG readily stabilized DNA droplets (Figures S5 and 2c). Importantly, no condensates formed when PEG 7.5k was added to NSs without SEs, whereas the presence of SEs led to an increase in *T*_p,t_ of up to approximately 15 °C (Figure S2). This indicates that PEG 7.5k does not directly induce the assembly of NSs but rather enhances SE-mediated interactions between NSs. Taken together, these results demonstrate that SE-mediated associative interactions between NSs act cooperatively with segregative interactions between PEG 7.5k and NSs in this system.

### PEG-Induced Partitioning of NSs into DNA Droplets

To assess the impact of PEG 7.5k on DNA droplets composed of NSs from a perspective distinct from thermal stability, the localization of NSs within the droplet interior (condensate phase) and exterior (dilute phase) was examined based on fluorescence microscopy (Nikon) images. Samples were observed at either 25 or 2 °C below the *T*_p,t_ after a 10 min incubation, both in the absence and presence of PEG 7.5k, to account for temperature-dependent droplet states and PEG-induced stability changes influencing NS localization. Consistent with previous report,^37^ gel-like structures were observed at 25 °C, a temperature far below *T*_p,t_, whereas distinct spherical and liquid-like droplets formed at a temperature 2 °C below *T*_p,t_ (Figure 3a). No contrast adjustment was applied, and all the microscopy conditions, including laser intensity, were maintained consistently across all measurements. Subsequently, the maximum fluorescence intensity within a central 600 × 600 pixel region was obtained from each image and compared. Upon the addition of PEG 7.5k, the maximum intensity increased by approximately 2-fold at both 25 and 2 °C below *T*_p,t_ (Figure 3b; the *y*-axis is shown on a logarithmic scale), indicating an increase in NS density within the condensate phase. Conversely, the minimum fluorescence intensity did not significantly change upon the addition of PEG 7.5k (Figure 3b). Although an increase in condensate density is expected to be accompanied by a decrease in NS concentration in the dilute phase, the low concentration in the dilute phase may have been below the detection limit of the microscopy.

**Figure 3:**
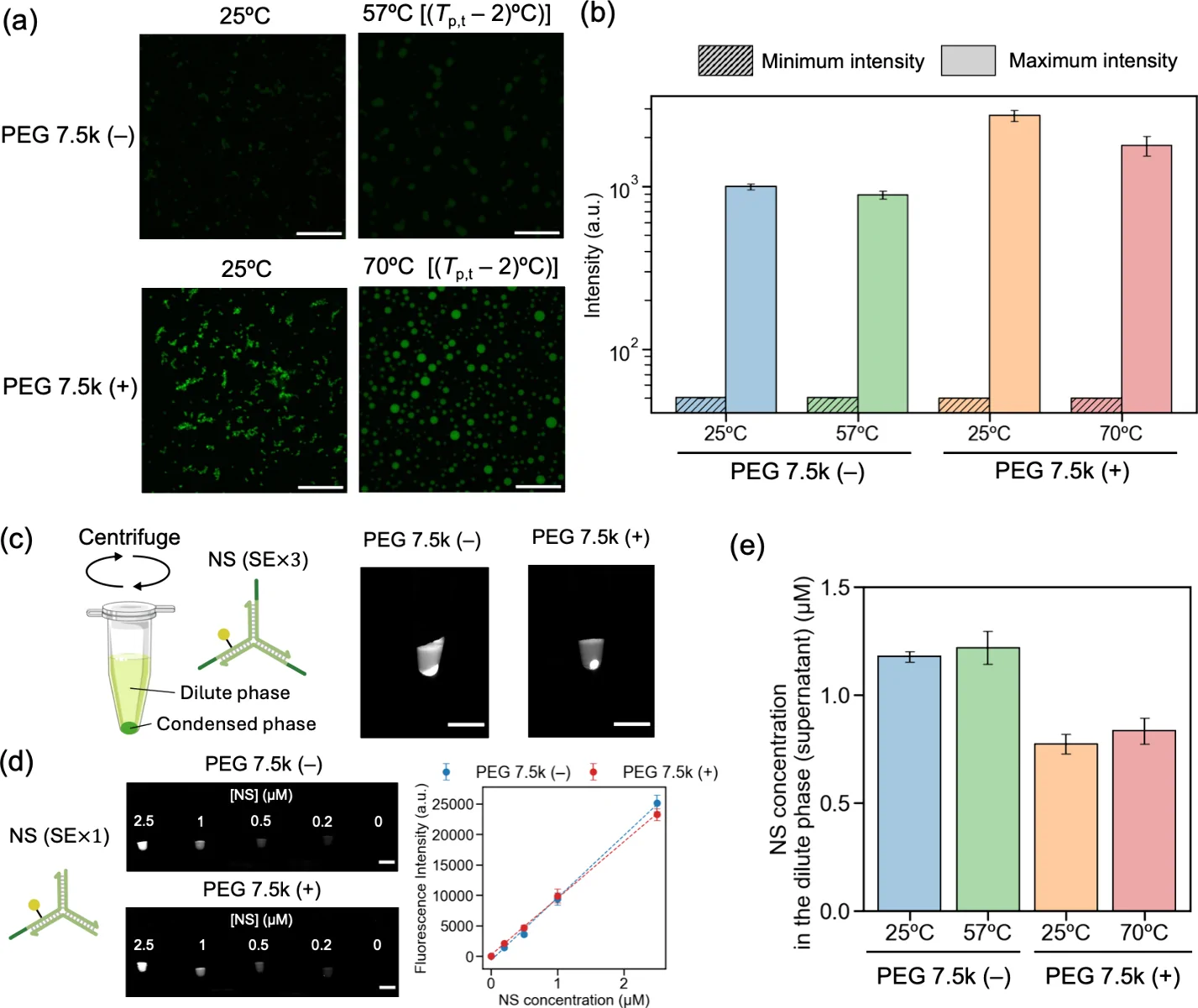
PEG 7.5k facilitates the partitioning of NSs into the condensate phase. (a) Microscopic images are shown without (−) and with (+) PEG 7.5k. These images were taken after a 10 min incubation at 25 °C or at a temperature 2 °C below the phase-separation temperature determined by turbidity measurement (*T*_p,t_). The confocal laser-scanning microscopy (Nikon) was used for imaging. (b) The maximum and minimum pixel intensities in each image shown in (a). (c) Fluorescence photo images of DNA droplets composed of NSs with three sticky ends (SEs) after centrifugation, both without (−) and with (+) PEG 7.5k. (d) Calibration curves (right) for quantifying NS concentration based on the fluorescence intensity of dispersed NS solutions (left), measured using a real-time PCR detection system. The calibration curves were obtained from dispersed NSs with only a single SE.(e) NS concentrations in the supernatant samples collected from (c), as determined from their fluorescence intensities using the calibration curves in (d). Error bars indicate standard errors (*n* = 9 for (b); *n* = 3 for (d); *n* = 3 for (e)). Scale bars: 50 μm in (a); 5 mm in (c) and (d).

To quantitatively evaluate the NS concentration in the dilute phase, the NS concentration in the supernatant obtained after centrifugation was measured using the fluorescence detection module of a real-time PCR system. Regardless of the presence of PEG, NSs containing three SEs exhibited clearly distinguishable condensate and dilute phases in fluorescence images after centrifugation (Figure 3c). In contrast, NSs with only a single SE did not form condensates and remained homogeneously dispersed in solution (Figure 3d), which was utilized to construct calibration curves to quantify the NS concentration from fluorescence intensity of a dispersed NS solution (Figure 3d). After centrifugation, samples were incubated at either 25 or 2 °C below *T*_p,t_ for 10 min, after which the supernatant was collected, and its fluorescence intensity was compared with the calibration curve to estimate the NS concentration in the dilute phase. Consequently, the addition of PEG 7.5k resulted in an approximately 1.7-fold decrease in the NS concentration in the dilute phase (Figure 3e). Collectively, these results demonstrate that PEG 7.5k facilitates the partitioning of NSs into DNA droplets at both 25 and 2 °C below *T*_p,t._, thereby enhancing their accumulation in the condensate phase.

### PEG-Induced Self-Organized Pattern Formation of DNA Droplets

The ability of PEG 7.5k to promote condensation of NSs with complementary SEs was employed to enhance an interaction that gather NSs with noncomplementary SEs. NS-A and NS-B were engineered with entirely noncomplementary sequences, including their SEs, resulting in the formation of immiscible A- and B-droplets, respectively (Figure 4a).^37^ Following thermal annealing, the NS samples were incubated at 60 °C for 30 min and subsequently observed with a confocal laser-scanning microscope. Under the condition of 0% (w/v) PEG 7.5k, A- and B-droplets were observed as independent droplets with different fluorescence (Figures 4b and S7a), showing different fluorescence intensity profiles from each droplet type. This independent droplet configuration was kept in the presence of 5% (w/v) PEG 7.5k (Figures 4b and S7a). Additionally, under 5% (w/v) PEG 7.5k conditions, the droplet diameter was reduced to approximately half of that observed at 0% (w/v) PEG 7.5k (Figure 4b). This finding suggests that the increase in solution viscosity induced by PEG 7.5k inhibits droplet diffusion and subsequent fusion, indicating that PEG 7.5k facilitates the size regulation of DNA droplets. Notably, at 10% (w/v) PEG 7.5k, A- and B-droplets exhibited contact patterns (Figures 4b and S7a), and fluorescence peaks from both droplets overlapped near the interface (Figure 4b). The concentration of 10% (w/v) PEG 7.5k corresponds to approximately twice its overlap concentration (Figure S4). Furthermore, at 20% (w/v) PEG 7.5k, the interface between A- and B-droplets became indistinct (Figure 4b). These behaviors agree with previously reported phenomena in which RNA condensates composed of two types of RNA nanostars form network-like patterns in the presence of PEG.^56^ By contrast, contact between A- and B-droplets was not observed in the presence of 10% (w/v) Dex 200k or Ficoll 400k (Figure S8). This result suggests that contact between immiscible droplets is related to the thermal-stabilizing effect of the crowding agent (Figures 1 and S8). Collectively, these results suggest that depletion interaction induced by PEG 7.5k gathered noncomplementary NS-A and NS-B and made A- and B-droplets contacted while avoiding fusion.

**Figure 4:**
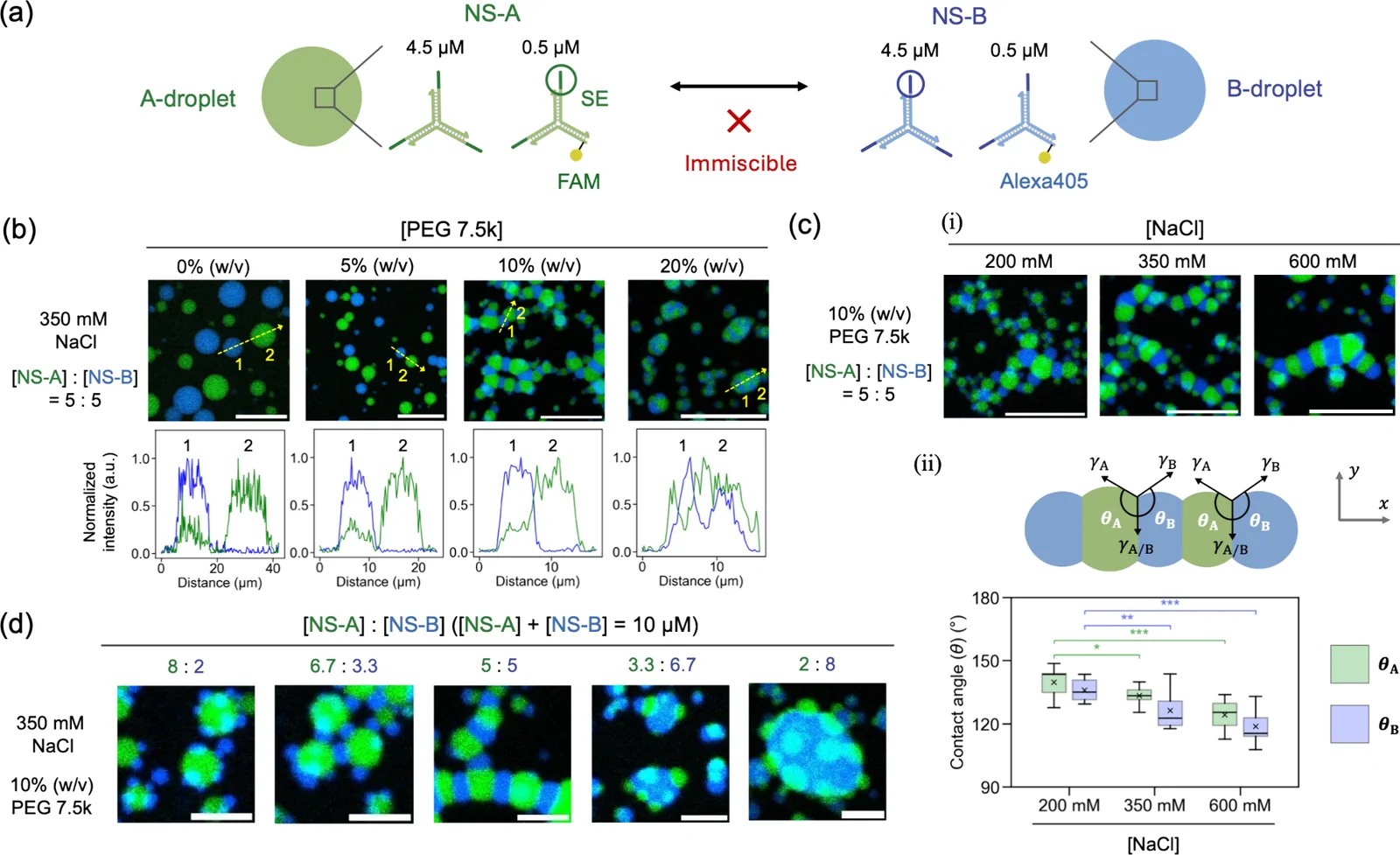
Network pattern formation induced by contact between two types of DNA droplets in the presence of PEG 7.5k at 60 °C. (a) The NS-A and NS-B each possessing sticky ends (SEs) which are mutually noncomplementary, forming two immiscible A- and B-droplets. 10% of NS-A and NS-B are labeled with 6-carboxyfluorescein and Alexa Fluor 405, respectively, where each lacks one SE. (b) PEG 7.5k concentration-dependent contact between A- and B-droplets. (c) NaCl concentration-dependent (i) pattern formation and (ii) box plot of the contact angle between A- and B-droplets (*n* = 9). One-tailed Welch’s *t* test was used for statistical analysis. Significance is indicated as *p* < 0.05 (*), *p* < 0.01 (**), and *p* < 0.001 (***) (d) Concentration-ratio-dependent pattern formation of NS-A and NS-B. Observations were performed using the confocal laser-scanning microscope (Nikon). Scale bars: 30 μm in (b) and (c), and 10 μm in (d).

The interactions between NS-A and NS-B upon contact in the presence of PEG 7.5k are anticipated to occur on a distance scale comparable to the Debye length, where electrostatic repulsive interactions arising from the negatively charged DNA become significant. To investigate this effect, changes in pattern formation were examined as a function of cation (Na^+^) concentration. At 200 mM NaCl, the interfacial area between A- and B-droplets was small, and both droplets maintained a relatively spherical morphology (Figures 4c-i and S7b). In contrast, at 350 and 600 mM NaCl, where electrostatic repulsion between DNA strands is screened more effectively, the interfacial area between A- and B-droplets appeared to increase (Figures 4c-i and S7b). The interfacial tensions at the droplet contact interfaces (γ_A_, γ_B_, and γ_A/B_) and the contact angles of A- and B-droplets (θ_A_ and θ_B_; 90° < θ_A_, θ_B_ < 180°) at the three-phase contact line (or referred to as the triple-line) are depicted in Figure 4c-ii. The interfacial tension equilibrium at a three-phase contact point is described by the “Neumann’s triangle”,^41,72–74^ and the following relationship holds for the y-component:

1 $$\gamma_{A/B}=\gamma_{A}\cos\left(\pi -\theta_{A}\right)+\gamma_{B}\cos\left(\pi -\theta_{B}\right)$$

Here, although NS-A and NS-B are immiscible, they are equivalent colloids; therefore, A- and B-droplets possess identical physical properties. Accordingly, assuming that γ_A_ = γ_B_ ≅ γ_0_ and θ_A_ = θ_B_ ≅ θ_0_, eq 1 can be rewritten as follows:

2 $$\cos\theta_{0}=\frac{-\gamma_{A/B}}{{2\gamma }_{0}}$$

The contact angles of both A- and B-droplets decreased toward 90° with increasing salt concentration (Figure 4c-ii). According to eq 2, as $\theta_{0}\to \frac{\pi }{2},\cos\theta_{0}\to 0$ . Increasing salt concentration screens the electrostatic repulsion between the NSs, leading to an increase in the solvent–NS interfacial tensions (γ_0_) of both NS-A and NS-B and a decrease in the interfacial tension between NS-A and NS-B (γ_A/B_), thereby reducing the contact angle (θ_0_). These results indicate that the wettability between the droplets can be controlled by the salt concentration. While previous studies have reported strategies to control wettability between immiscible DNA condensates through the design of cross-linking linker DNA^41^ or electrostatic interactions with cationic molecules, including cationic peptides (e.g., oligolysine at 150 mM NaCl,^75^ poly-l-lysine at 1 M NaCl^76^) and cationic lipids at water–oil interfaces,^77^ the enhanced wettability observed in this system is attributed to the cooperative effects of PEG 7.5k–induced depletion interactions and salt-dependent electrostatic screening.

In systems comprising immiscible droplets of synthetic polymers, it has been reported that altering the concentration ratio of the constituent components results in distinct pattern formation behaviors.^78^ In this study, the total concentration of NS-A and NS-B was maintained at 10 μM, while their concentration ratio was systematically adjusted (8:2, 6.7:3.3, 5:5, 3.3:6.7, and 2:8). Under conditions with a biased concentration ratio, a relatively large droplet of higher-concentration NSs was surrounded by multiple smaller droplets of the lower-concentration NSs (Figures 4d and S7c). When NS-A and NS-B were present at equal concentrations, a network pattern of string-like structures of alternately contacted A- and B-droplets emerged (Figures 4d and S7c). This difference could be attributed to the differences in the initial droplet number density and fusion frequency. Specifically, the number of droplets composed of the higher-concentration NS increased, resulting in frequent collisions, promoting fusion to form fewer larger droplets. The number of droplets composed of lower-concentration NS was smaller, and the droplets could not collide, thereby maintaining the smaller droplets. When both components are present in equal concentrations, both droplet phases undergo contact and fusion at comparable frequencies, forming droplets of similar size. The further fusion of A-droplets is inhibited by immiscible B-droplets, leading to alternately contacted A- and B-droplets, and vice versa.

In the final analysis, the influence of PEG 7.5k was assessed on three distinct NS motifs—NS-A, NS-B, and NS-C—which correspondingly form immiscible A-, B-, and C-droplets (Figure 5a).^46,53^ Under conditions of 0% (w/v) PEG 7.5k, A-, B-, and C-droplets were observed as separate entities, with distinct fluorescence intensity profiles clearly demarcated by labeling NS-A, NS-B, and NS-C with different fluorescent dyes (Figures 5b and S9). At a concentration of 5% (w/v) PEG 7.5k, no droplet contact was observed, similar to the two-droplet system (Figure 4b), and the droplet diameters were reduced to approximately half of those observed in the absence of PEG (Figures 5b and S9). At 10% (w/v) PEG 7.5k, droplets of all three types were in contact; however, despite incubation at 60 °C for 30 min, the droplet sizes remained smaller, and the interfaces were less distinguishable (Figure 5b and S9) compared to the two-droplet system (Figure 4b). This observation suggests that the presence of the third type of droplet inhibits the fusion of the other two droplets. To facilitate fusion, the incubation temperature was increased to 70 °C for 30 min. Consequently, droplet sizes increased, and multicellular-like structures comprising three droplet phases were observed (Figures 5b and S9). The resulting patten is analogous to a proper 3-coloring in graph theory, where droplets of the same type were not adjacent to each other (Figures 5c). Since the three NSs are present in equal concentrations, each droplet phase comes into contact and fusion with the others at roughly the same frequency, forming droplets of similar size. The fusion of A-droplets is inhibited by the immiscible B- and C-droplets, causing B- and C-droplets to alternate in a circular pattern around the A-droplets (Figure 5c–ii). The same applies to B- and C-droplets (Figure 5c–iii and c–iv).

**Figure 5:**
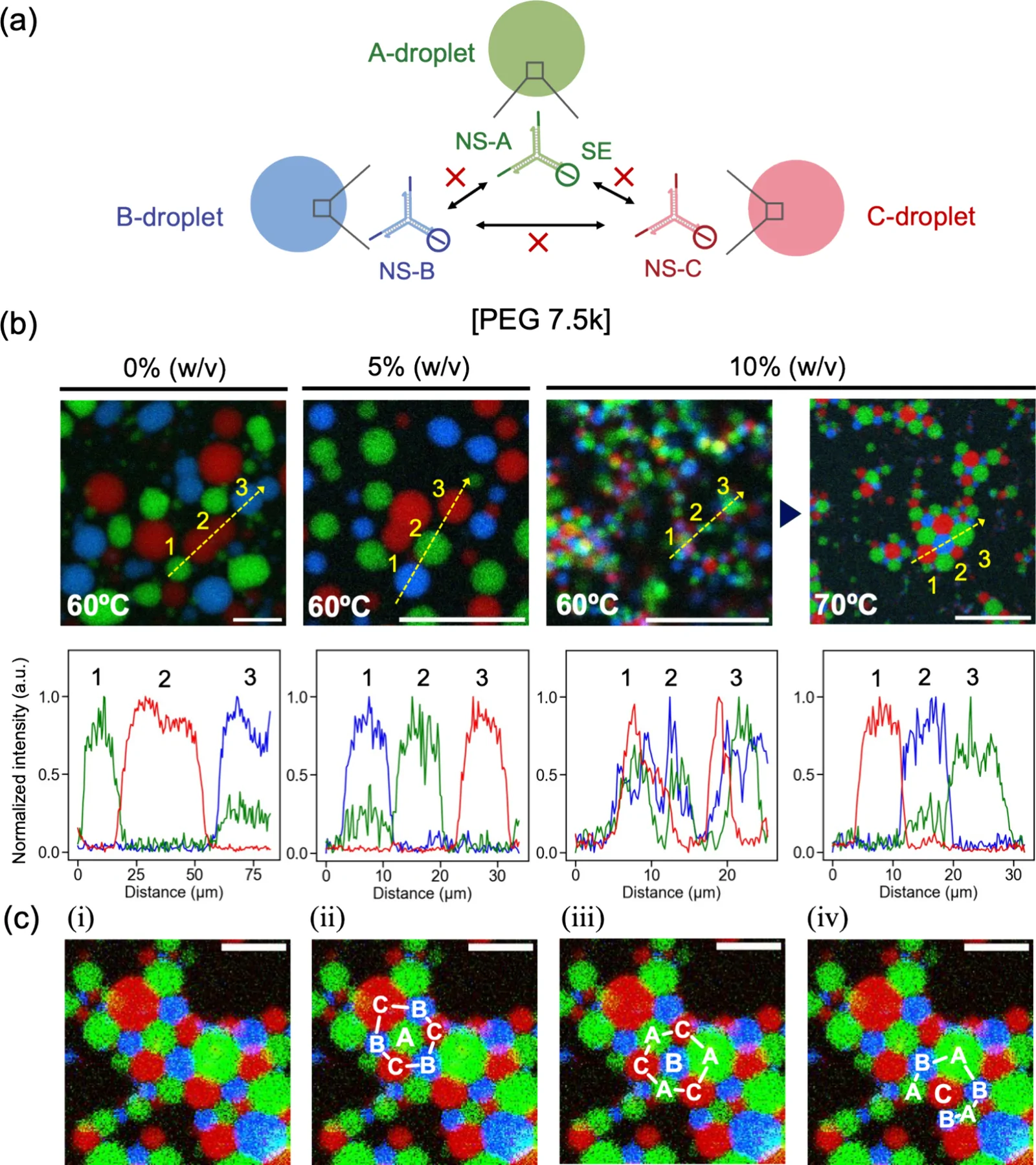
PEG 7.5k induced a networked pattern among three distinct types of DNA droplets. (a) NS-A, NS-B, and NS-C, each with mutually noncomplementary sticky ends (SEs), form immiscible A-, B-, and C-droplets. 10% of NS-A, NS-B and NS-C are labeled with 6-carboxyfluorescein, Alexa Fluor 405 and Cyanine5, respectively, each lacking one SE. PEG 7.5k concentration-dependent (b) pattern formation of A-, B-, and C-droplets and fluorescence intensity line profiles. (c) Pattern formation by three droplets in a proper 3-coloring, where adjacent droplets exhibit distinct colors. The observations were performed at 70 °C in the presence of 10% (w/v) PEG 7.5k. The confocal laser-scanning microscope (Nikon) was used for observations in (b) and (c). Scale bars: 30 μm in (b) and 10 μm in (c).

## Conclusions

In this study, we examined the impact of macromolecular crowding on liquid-like DNA condensates formed through the SE hybridization of NSs, utilizing representative polymeric crowding agents: PEG, dextran, and Ficoll. Among these crowding agents, PEG was found to be the most effective in stabilizing DNA droplets, significantly increasing their phase-separation temperature compared to dextran and Ficoll. This effect was strongly dependent on the MW of PEG, with PEG 7.5k enhancing both droplet formation and thermal stability, whereas low-MW PEGs (≤1k) led to droplet destabilization. This behavior may arise from the weak depletion effect of low-MW PEGs (≤1k), because these PEGs are expected to remain below the overlap concentration (c*) under the 10% (w/v) condition, i.e., *c*/c* < 1. However, the limited stabilization observed for low-MW PEGs (≤1k) even at *c*/c* < 1 suggests that depletion alone cannot fully explain the PEG-MW dependence. Reduced water activity, which may disfavor DNA hybridization that requires proper hydration, and possible kinetic or viscosity effects at high PEG concentrations may also contribute. In addition, experimental validation of PEG exclusion^79^ using fluorescently labeled PEG would help elucidate the mechanism of the PEG-induced depletion effect on DNA droplets. These findings suggest that the stability of DNA droplets is governed by the cooperative interplay between SE-mediated associative interactions among NSs and PEG-induced segregative interactions.

In addition to enhancing thermal stability, PEG 7.5k facilitated the partitioning of NSs into the condensate phase, thereby increasing the condensate density and decreasing the NS concentration in the dilute phase. Furthermore, the PEG-induced depletion interaction enabled tunable contact, wettability, and network-like pattern formation between immiscible DNA droplets. This pattern formation was not observed in the presence of Dex or Ficoll for the size and the concentration in the case of the experiments, suggesting that exploring the crowding effects of general polymers other than PEG for the pattern formation will be the next challenge. This PEG-mediated spatial organization was further modulated by PEG concentration, salt concentration, and the ratio of NS compositions, and could be extended to multicomponent systems comprising three immiscible droplets. Quantitative comparison with other wettability-control strategies, such as cross-linking linker DNA^41^ and cationic molecules,^75–77^ would help further characterize PEG-mediated droplet morphology control.

The influence of PEG on DNA has been examined at the molecular level, where the elongation and compaction of DNA^80^ have been elucidated from single-molecule or nanoscale perspectives. Concurrently, microscale structural organization has been reported including the formation of liquid-crystalline phases associated with DNA elongation,^81^ as well as phase segregation between PEG and DNA under confined conditions.^60^ More recently, the addition of PEG has been shown to induce microscale volume phase transition of DNA condensates composed of NSs.^82^ In this study, we demonstrated that PEG-induced depletion interaction, a nanoscale effect, contributes to tens-of-micrometer-scale phenomena, such as pattern formation of DNA droplets and material properties

The self-organized pattern formation of DNA condensates shown here suggests a potential foundation for morphological control and spatiotemporal regulation of reaction fields. By adjusting intercondensate interactions and contact geometries, reactions can be localized and propagated in space and time, making this system valuable for bottom-up synthetic biology, including applications in artificial cells and molecular robotics. This system can be extended to diverse pattern formations by integrating it with microfluidic platforms. We have recently observed that uniform NS-based DNA hydrogels localize at the interface of a dextran/PEG aqueous two-phase system, forming an aqueous three-phase system.^83^ The pattern formation driven by PEG-induced depletion interactions acting on NS as valency-controllable colloids can be extended to condensates based on peptides, proteins, and synthetic polymers, providing a general design principle for programmable soft matter.

## Materials and Methods

### Material

Sodium chloride (191–01665), polyethylene glycol 600 (168–09075; molecular weight (MW) ≈ 0.6k; hereafter referred to as PEG 0.6k), polyethylene glycol 1000 (165-09085; MW ≈ 1k; PEG 1k), polyethylene glycol 4000 (162-09115; MW ≈ 3k; PEG 3k), polyethylene glycol 6000 (169-09125; MW ≈ 7.5k; PEG 7.5k), dextran 200k (041-22612; MW ≈ 200k; Dex 200k) and bovine serum albumin (019-15123; BSA) were purchased from FUJIFILM Wako Pure Chemical Corp. (Osaka, Japan). Polyethylene glycol 8000 (195445; MW ≈ 8k; PEG 8k) was obtained from MP Biomedicals, LLC (Santa Ana, CA, USA). Ficoll 400 (F2637-5G; MW ≈ 400k; Ficoll 400k) was obtained from Sigma-Aldrich (St. Louis, MO, USA). 1 M Tris-HCl pH 8.0 (15568025) was acquired from Thermo Fisher Scientific (Tokyo, Japan). Mineral oil (23334-85) was purchased from Nacalai Tesque, Inc. (Kyoto, Japan). Glass slides (30 × 40 mm, 0.17 mm thick; C030401) and coverslips (18 × 18 mm, 0.17 mm thick; C218181) were purchased from Matsunami Glass (Kishiwada, Japan). Double-sided tapes with thicknesses of 30 μm (707#4) and 1 mm (T-4613) were supplied by Teraoka Seisakusho Co., Ltd. (Tokyo, Japan) and Nitoms, Inc. (Tokyo, Japan), respectively. The DNA sequences listed in Table S1 were purchased from Eurofins Genomics (Tokyo, Japan). The DNA strands labeled with fluorescent modifications were of high-performance liquid chromatography (HPLC) grade, while the unlabeled strands were of oligonucleotide purification cartridge (OPC) grade. Ultrapure water was produced using a Direct-Q UV system (Merck Millipore, MA, USA).

At concentrations of around 10% (w/w) range or lower, PEG (M*W* ≤ 8k),^84^ Dex 200k^85^ and Ficoll 400k^86^ solutions have densities close to ∼1 g/cm^3^; thus, values in the ‘% (w/v)’ unit are assumed to be the same as those in the ‘% (w/w)’ unit.

### Measurement of Phase-Separation Temperatures by Microscopic Observation (*T*_p,m_)

DNA strands forming NS-A and crowding agents (PEG 7.5k, Dex 200k, or Ficoll 400k) were mixed in a buffer consisting of Tris-HCl (pH 7.5) and NaCl, and their concentrations are listed in Table S2. The observation chamber was prepared using a previously reported method.^37^ Glass slides and coverslips were immersed in 5% (w/v) BSA solution in 20 mM Tris-HCl (pH 7.5) for over 30 min. After BSA coating, slides and coverslips were rinsed with ultrapure water and dried. The BSA-coated slides and coverslips were assembled using 30 μm double-sided tape. The DNA sample solution was introduced between the coated slides and coverslips. The coverslip edges were sealed with manicure paste. To prevent evaporation during observation, mineral oil was added using a 1 mm double-sided tape bank. Samples were visualized using a fluorescent microscope (IX-71, Olympus, Tokyo, Japan) equipped with a spinning-disk confocal system (CSU-X1, Yokogawa, Tokyo, Japan), an EM CCD camera (iXon X3, Andor), and a stage heater (10021-PE120 system, Linkam, Fukuoka, Japan). Samples containing 6-carboxyfluorescein (6-FAM)–labeled DNA were excited at 473 nm.

The chamber was incubated at 85 °C for 3 min using the stage heater. Temperature decreased at 1 °C per min, with observations at each interval. To determine *T*_p,m_, droplets were quantified using ImageJ’s “Analyze Particles”. Images were binarized using a 99th percentile threshold. Droplets with an area of at least 1 μm^2^ were counted, and *T*_p,m_ was defined when droplet count reached five or more. Given an observation area of 136.7 × 136.7 μm^2^ and an effective observation depth of 1.8 μm (corresponding to an observation volume of ∼34 pL), counting droplets with a projected area of ≥1 μm^2^ corresponds to counting droplets with volumes of ≥∼1.8 fL.

### Measurement of Phase-Separation Temperatures by Turbidity Observation (*T*_p,t_)

DNA strands forming NS-A and PEG 0.6k, 1k, 3k, 7.5k, and 8k were mixed in a buffer consisting of Tris-HCl (pH 7.5) and NaCl, with their respective concentrations detailed in Tables S3 and S4. Absorbance at 600 nm (i.e., turbidity) of the solutions at temperatures from 99 to 25 °C was measured on a UV–visible spectrophotometer with a micro-8-well cell block (V-630Bio, JASCO, Tokyo, Japan) equipped with a Peltier temperature controller at a temperature changing rate of – 1 °C/min. *T*_p,t_ was defined as an initial point of temperature where the absorbance first began to up as the sample was cooled from 99 to 25 °C.

### Intensity Ratio Analysis

Oligonucleotides forming NSs and PEG 7.5k were combined in a buffer composed of Tris-HCl (pH 7.5) and NaCl, following the concentration outlined in Table S5. The sample was heated to 85 °C for 3 min and then cooled to 25 °C at a rate of – 1 °C/min using a thermal cycler (Mastercycler nexus X2, Eppendorf, Germany). BSA-coated glass slides were prepared as described above. Double-sided tape (1 mm thick) with 5 mm-diameter holes was affixed to the BSA-coated glass. DNA samples were introduced into the chamber holes and covered with mineral oil to prevent evaporation. Samples were visualized using a confocal laser scanning microscope (AX R, Nikon, Tokyo, Japan) and stage heater. Samples containing fluorescein-labeled DNA were excited at 488 nm. After incubation for 10 min at either 2 °C below *T*_p,t_ or at 25 °C, the observation was conducted.

Intensity ratio analysis followed established methodology^75^ using Fiji (ImageJ).^87^ Briefly, images were processed using a median filter with a five-pixel radius. Maximum and minimum intensities were determined within a 600-pixel square center. The maximum and minimum intensities were obtained. Images from three experiments were captured using a 40× objective lens, 8× frame averaging, 0.5 μs pixel dwell time, 10% laser power, and 50% camera gain.

### Estimation of the Dilute-Phase Concentration

The concentrations of NSs in the dilute phase during droplet and gel states were estimated by measuring fluorescence intensity of the supernatant after centrifugation, which precipitated DNA droplets and gels, as described previously.^37^ A calibration curve relating fluorescence intensity to concentration was constructed using fluorescein-labeled NSs with a single SE, which do not form condensates. Samples, prepared according to Table S6, were annealed in PCR tubes with the thermal cycler, cooling from 95 to 25 °C at a rate of 1 °C per min. The annealed NSs with a single SE were diluted to final concentrations of 2.5, 1.0, 0.5, 0.2, and 0 μM using the original dissolution buffer. Fluorescence intensity was measured using a real-time PCR detection system (CFX Connect, Bio-Rad, CA, USA). NSs with three SEs, which form condensates, were annealed using procedures outlined in Table S5. The solution volume was 20 μL. The PCR tubes were centrifuged at 20,000G and 25 °C for 20 min. The phase-separated NSs, consisting of condensed and dilute phases, were then verified using a fluorescent image analyzer (FLA-5100, FUJIFILM Corporation, Tokyo, Japan). To evaluate dilute-phase concentrations, temperatures were set to 2 °C below the *T*_p,t_ or to 25 °C. After 10 min incubation, 5 μL of supernatant was collected, diluted to 10 μL with buffer, and fluorescence intensity was measured. Dilute-phase concentrations were estimated using the calibration curve.

### Observation of Self-Organized Pattern Formation in DNA Droplets

As described in Tables S7–S9, samples were prepared in PCR tubes and then annealed at 85 °C for 3 min using the thermal cycler, followed by cooling to 25 °C at a rate of – 1 °C/min. The samples were then placed into observation chambers as described previously. After being incubated for 30 min at 60 or 70 °C on the stage heater, the samples were observed with the confocal laser scanning microscope (Nikon). DNA samples labeled with the fluorescent dyes 6-FAM, Alexa Fluor 405, and Cyanine5 (Cy5) were observed using excitation wavelengths of 488, 405, and 561 nm, respectively. The image intensities were normalized through min–max normalization according to the following equation:

3 $$I_{\mathrm{norm}}=\frac{I-I_{\min}}{I_{\max}-I_{\min}}$$

where *I* denotes the original image intensity, and *I*_min_ and *I*_max_ represent the minimum and maximum intensity values within the image, respectively.

### Contact Angle Analysis

In accordance with a previously reported method^74^ with slight modifications, a custom Python script was employed to analyze the contact angles of the A- and B-droplet phases, which were formed by the assembly of NS-A and NS-B, respectively. Confocal laser-scanning microscope (Nikon) images were cropped using Fiji (ImageJ)^87^ to isolate the regions of interest. Figure S6 shows the essential steps in image processing of typical droplets with interfaces. For each fluorescence channel, the intensities were standardized using the first and 99.fifth percentiles, then smoothed with a Gaussian filter, and converted to binary using Otsu’s method. The outlines of the droplets were derived from the binary masks. To reduce distortion caused by wetting, pixels near the A–B droplet interface were excluded from the contour before performing least-squares circular fitting on both the A and B droplets. The triple point was identified as the end point of the interface that minimized the total distance to the perimeter point sets of the A-droplet and B-droplet phases. The tangents to the fitted circles for both droplet phases were calculated at the triple point. The interfacial tangent was determined from the local interface contour around the triple point by applying principal component analysis (PCA) to the interface points and approximating the interface as a straight line; this linear approximation was applicable to all analyzed images near the triple point. Contact angles were measured as the larger angle between the interfacial tangent and each droplet tangent.

## Supplementary Material



## Notes

Definition of overlap concentration (Figure S1); fluorescence microscopy images and absorbance curves showing PEG and SE effects (Figure S2); fluorescence microscopy images with different PEG MWs (Figure S3); phase-separation temperature at overlap concentration of PEG (Figure S4); NaCl- and PEG 7.5k concentration-dependent absorbance curves (Figure S5); image segmentation workflow (Figure S6); wide-field fluorescence microscopy images showing pattern formation (Figures S7 and S9); effects of crowding agents on A/B-droplet contact-pattern formation (Figure S8); oligonucleotide sequences (Table S1); experimental concentration tables (Tables S2–S10) (PDF)

## ABBREVIATIONS

LLPS: Liquid–liquid phase separation
PEG: Polyethylene glycol
Dex: Dextran
NS: 3-arm DNA nanostar
SE: Sticky end
MW: Molecular weight
*R*_g_: Radius of gyration
*c**: Overlap concentration
*T*_p_: Phase-separation temperature
*T*_p,m_: Phase-separation temperature determined by microscopic observation
*T*_p,t_: Phase-separation temperature determined by turbidity measurement
