# Supplementary material for: Molecular Crowding Stabilizes DNA Coacervate Droplets and Generates Self-Organized Characteristic Patterns
Source: JACS Au. 2026 Jul 27;6(8):4667–78. doi: 10.1021/jacsau.6c00746 (PMC13507995; doi:10.1021/jacsau.6c00746)
Supplement: Supplementary file 1 [file au6c00746_si_001.pdf]

Supporting Information

Molecular Crowding Stabilizes DNA Coacervate Droplets and Generates Self-Organized Characteristic Patterns

Naoki C. Yoshida[†]*, Mayu Shono*[‡]*, Kenichi Yoshikawa*[§]*, and Masahiro Takinoue*[*, †, ¶, ǀ]

[†]Department of Life Science and Technology, Institute of Science Tokyo, 4259 Nagatsuta-cho, Midori-ku, Yokohama, Kanagawa 226-8501, Japan

[‡]Komaba Institute for Science, Graduate School of Arts and Sciences, The University of Tokyo, Komaba 3-8-1, Meguro, Tokyo, 153-8902, Japan

[§]Faculty of Life and Medical Sciences, Doshisha University, Kyoto, 610-0394, Japan

[¶]Laboratory for Chemistry and Life Science, Institute of Integrated Research, Institute of Science Tokyo, 4259 Nagatsuta-cho, Midori-ku, Yokohama, Kanagawa 226-8501, Japan

[ǀ]Research Center for Autonomous Systems Materialogy (ASMat), Institute of Integrated Research, Institute of Science Tokyo, 4259 Nagatsuta-cho, Midori-ku, Yokohama, Kanagawa 226-8501, Japan

1. **Supplementary Figures**

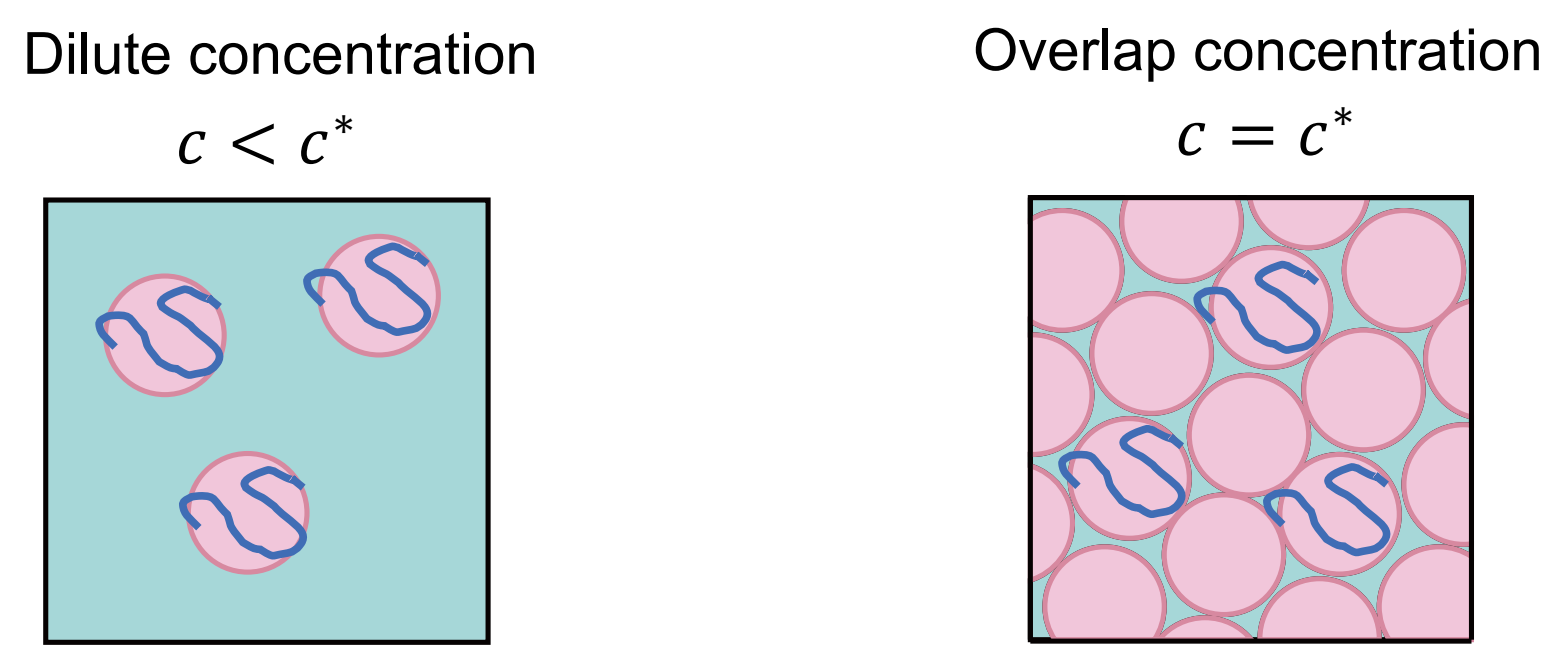

Figure S1. The overlap concentration (c^*), is defined as the polymer concentration at which individual polymer coils begin to overlap. Below this concentration ($c < c^*$), polymer chains remain isolated, corresponding to the dilute regime.

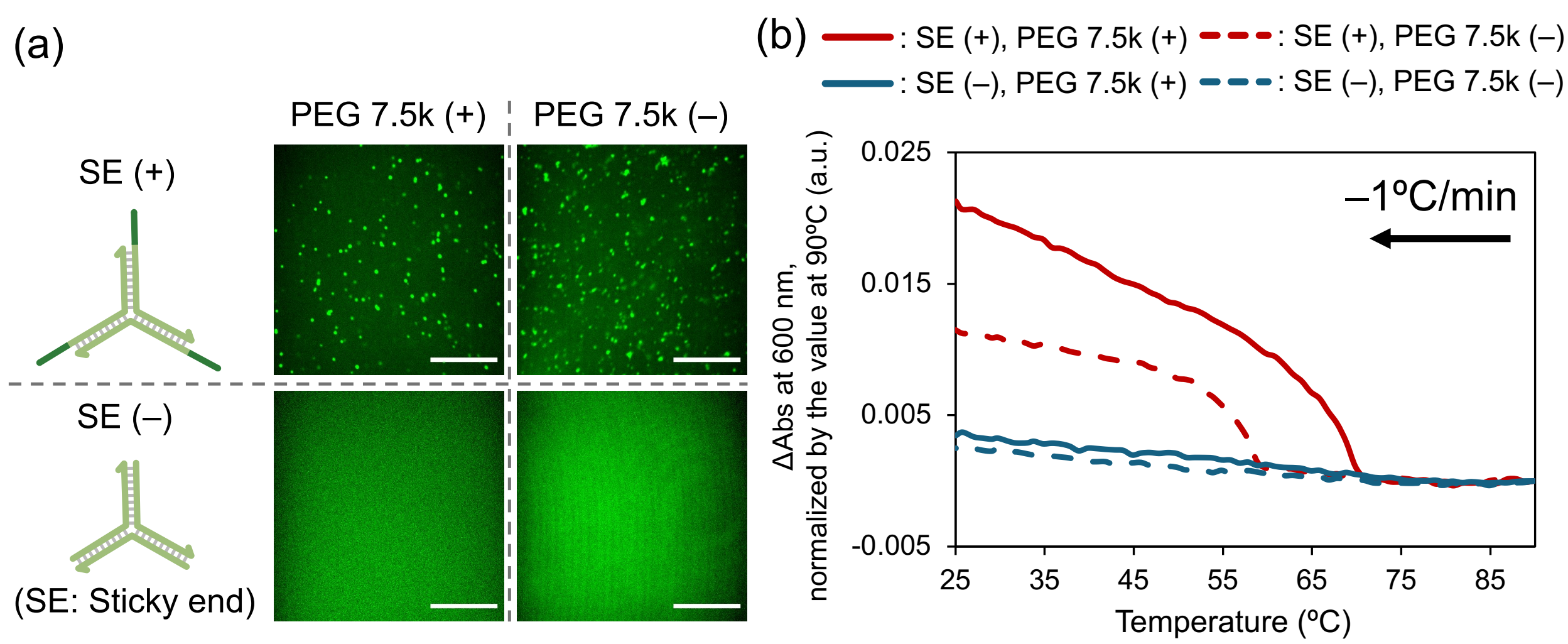

Figure S2. (a) Fluorescence microscopy images (Olympus) at 25 °C and (b) absorbance curves at 600 nm for NSs during cooling. Measurements were performed for NSs with (+) or without (–) sticky ends in the absence (–) or presence (+) of 10% (w/v) PEG 7.5k. [NS] = 5 µM. Scale bars: 100 µm.

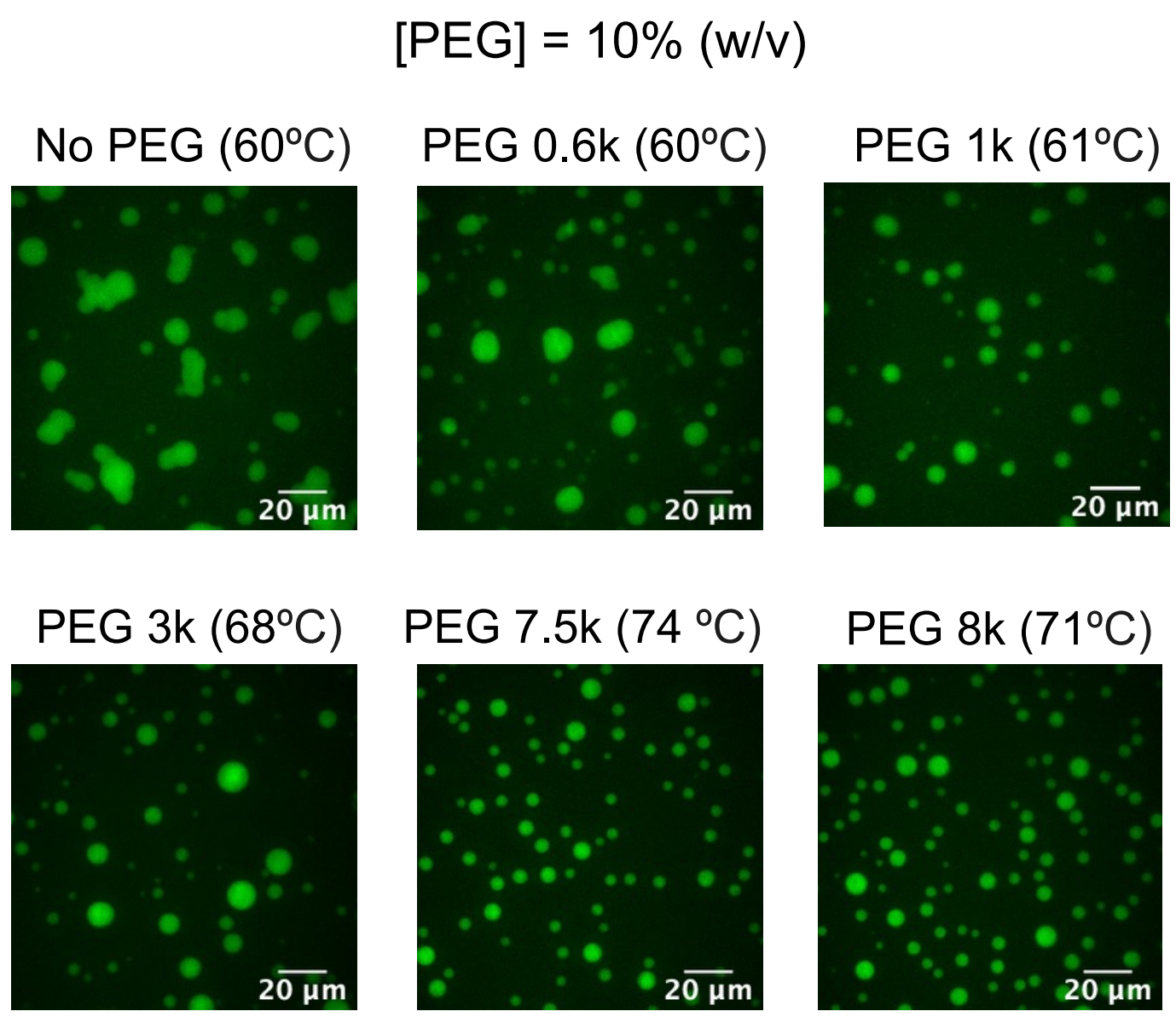

Figure S3. Fluorescence microscopy (Olympus) images of DNA droplets consisting of NSs, observed in the absence and presence of 10% (w/v) PEG with varying molecular weights.

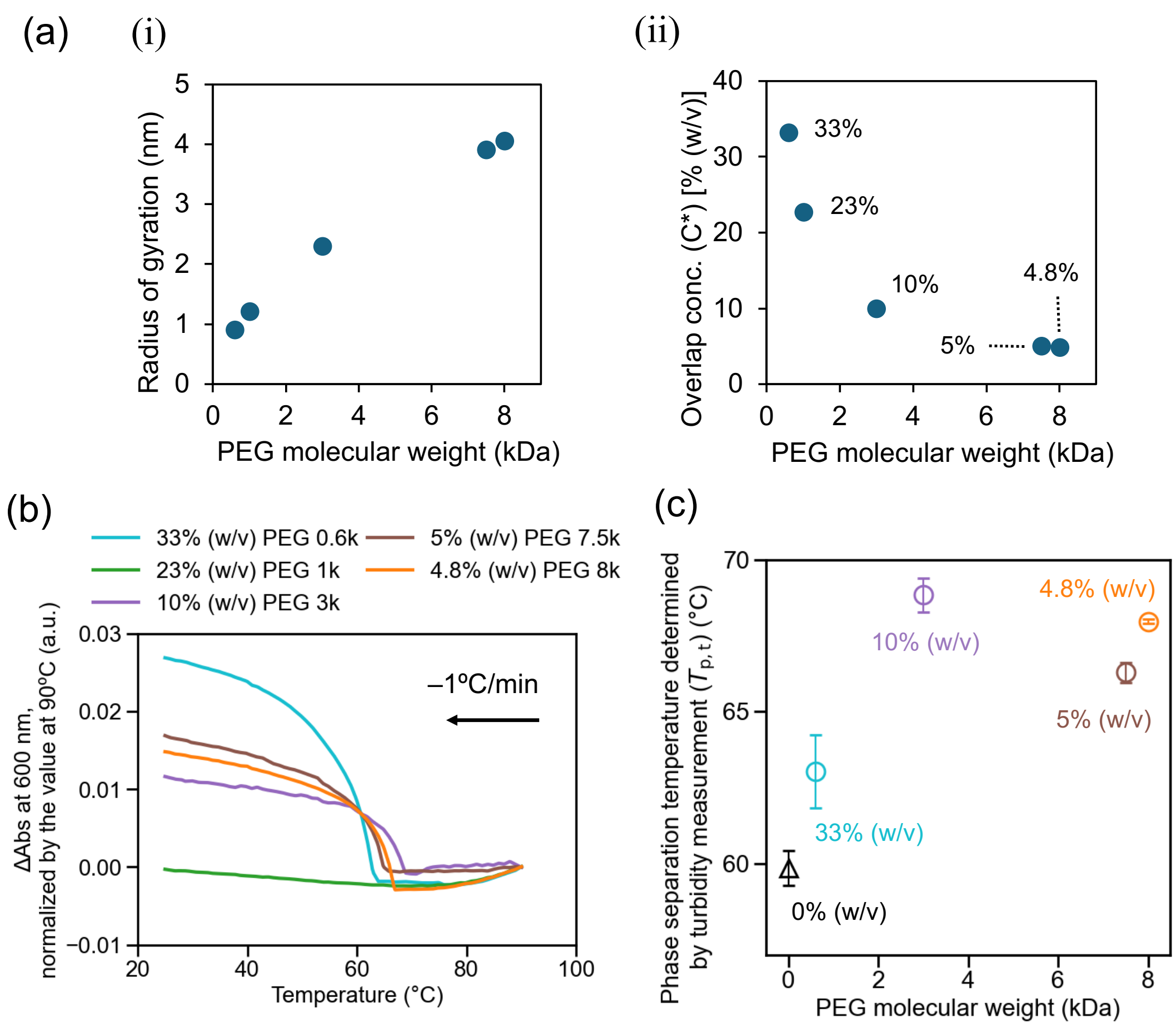

Figure S4. Phase-separation temperature at the overlap concentration. (a) PEG molecular weight (MW)-dependent (ii) radius of gyration (R_g) is given by $R_g = 0.0215 \cdot M_w{}^{0.583}$ (1) and (iii) the overlap concentration (c^*) is estimated as $c^* = \frac{M_w}{4/3\pi R_g{}^3 N_A}$, where N_A is Avogadro's constant. (b) Absorbance curves at 600 nm recorded during cooling and (c) phase separation temperatures of NSs in the presence of PEG with varying MWs at their respective c^*. The absence of a discernible transition point at 23% (w/v) PEG 1k (b) is likely attributable to the high viscosity of the solvent, which impeded the formation of DNA droplets necessary for scattering light at 600 nm.

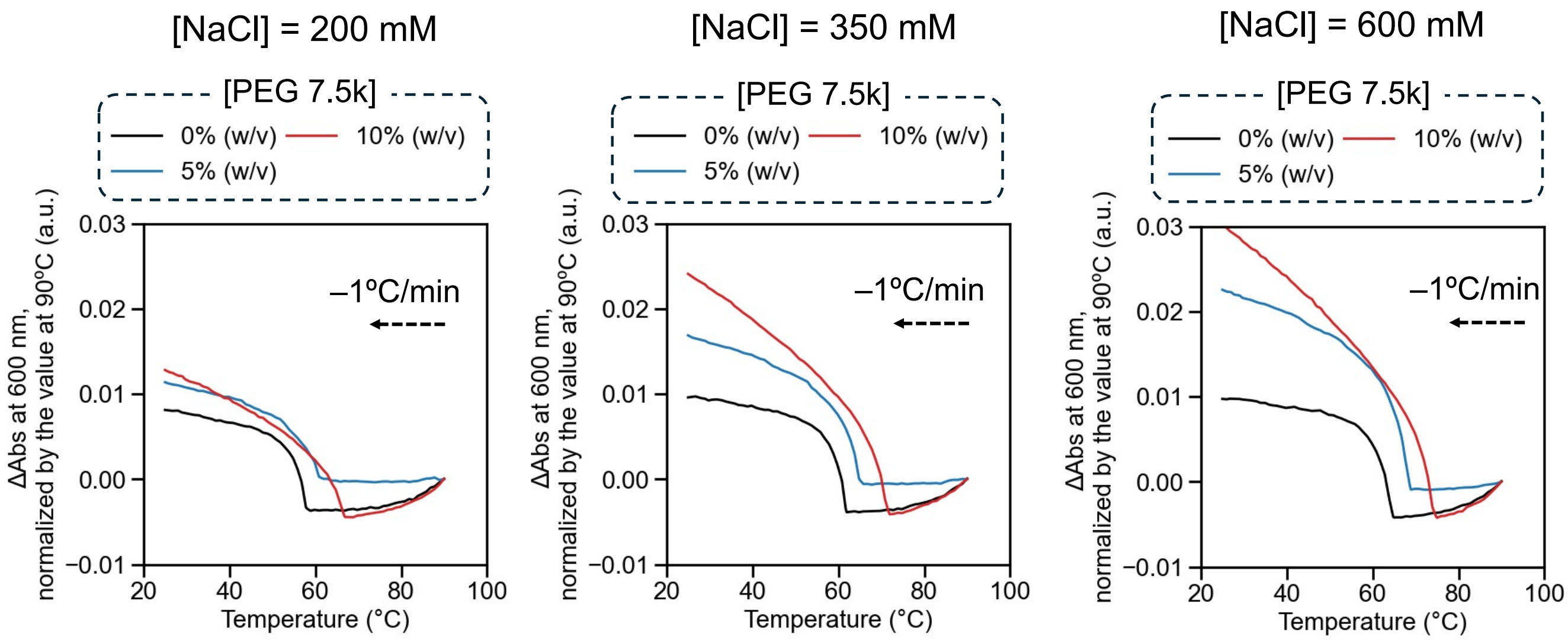

Figure S5. Temperature-dependent absorbance at 600 nm during the cooling process for NS solutions at different NaCl and PEG 7.5k concentrations.

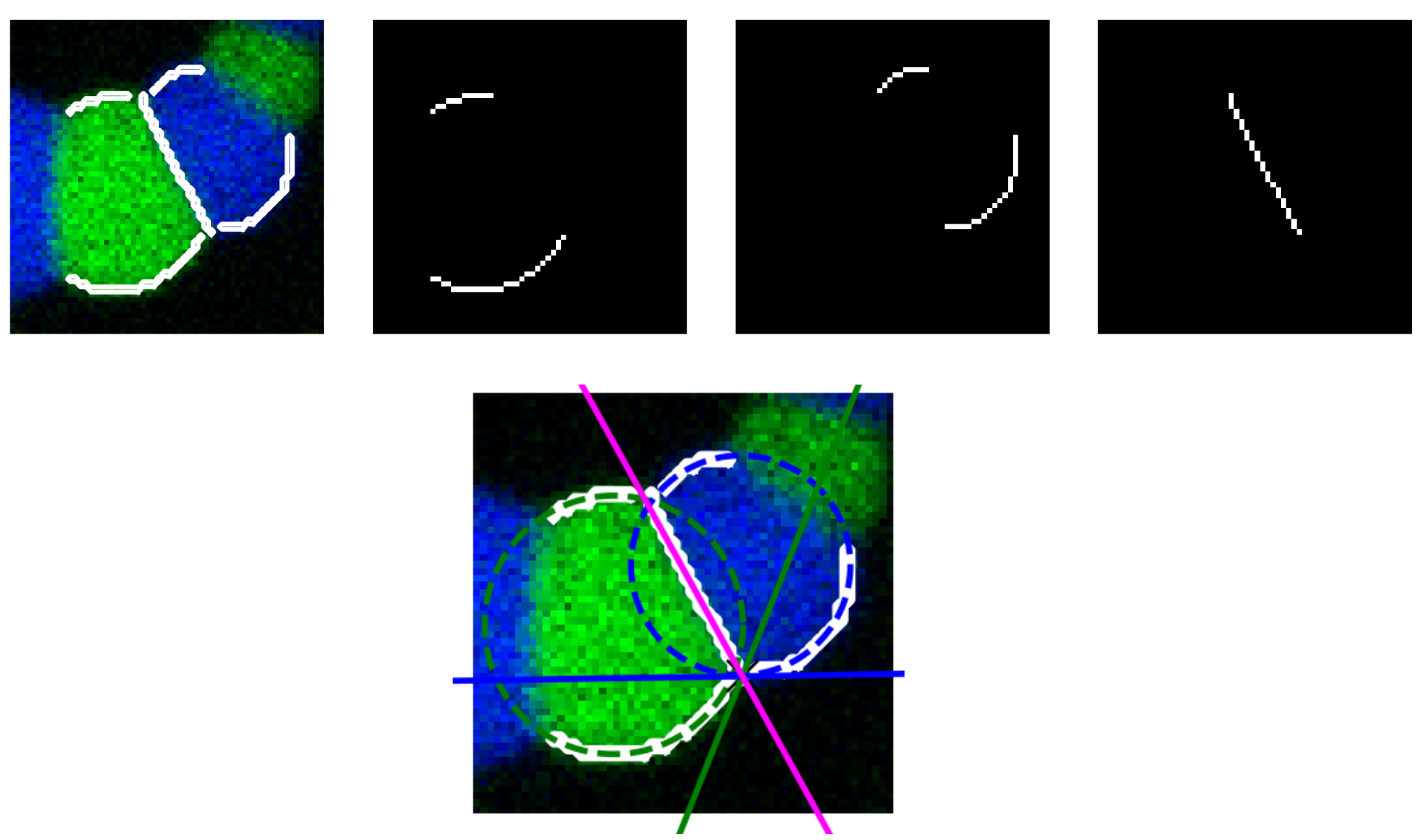

Figure S6. The image segmentation workflow employed to generate masks for the calculation of contact angles in droplets with multiple interfaces.

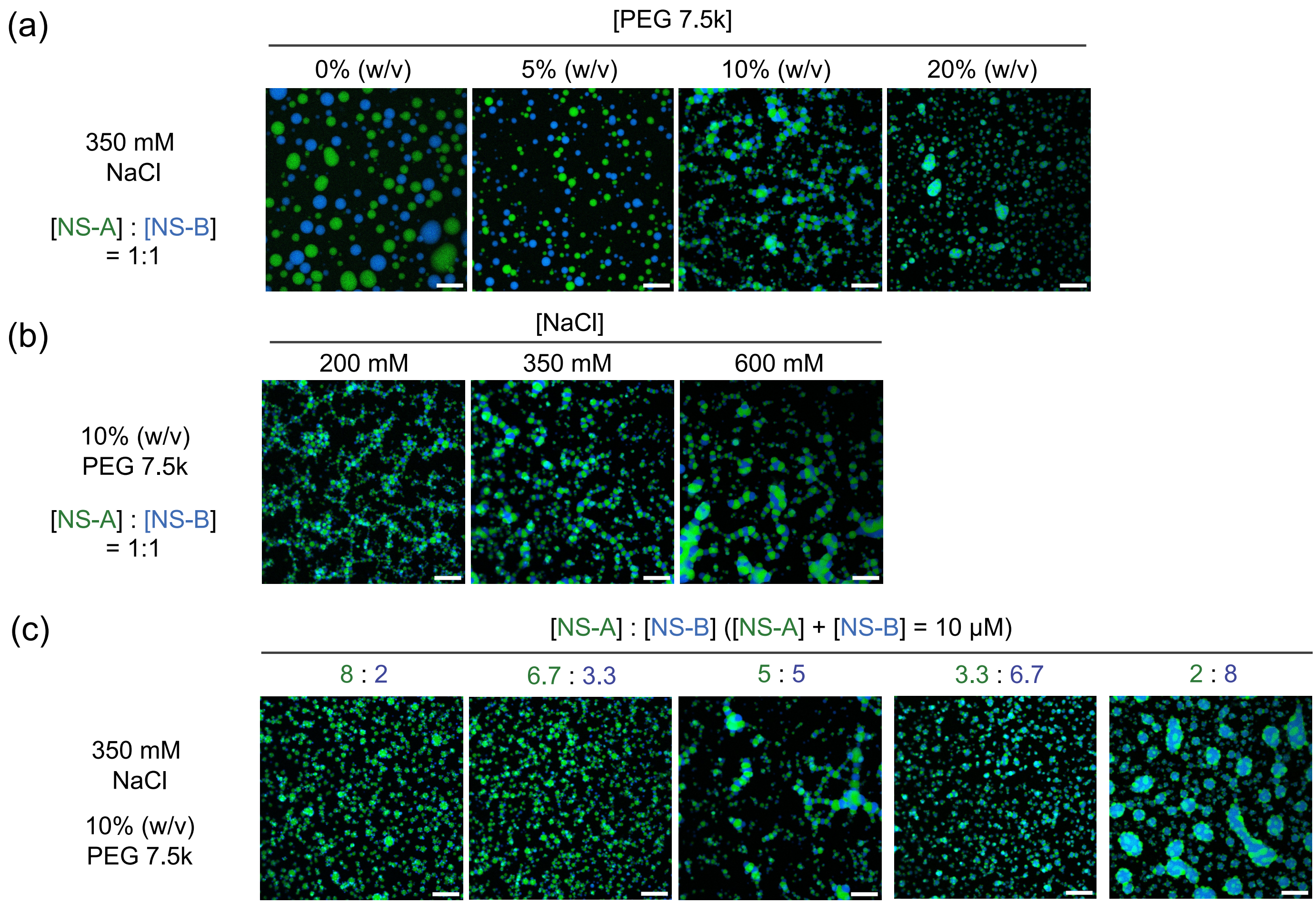

Figure S7. Wide-field fluorescence microscopy images showing the self-organized pattern formation of A- and B-droplets, which are composed of NS-A and NS-B, respectively. Representative images show (a) the effect of varying PEG 7.5k concentration, (b) the influence of NaCl concentration, and (c) the impact of the concentration ratio of NS-A and NS-B. The confocal laser-scanning microscope (Nikon) was used for observation. Scale bars: 30 μm.

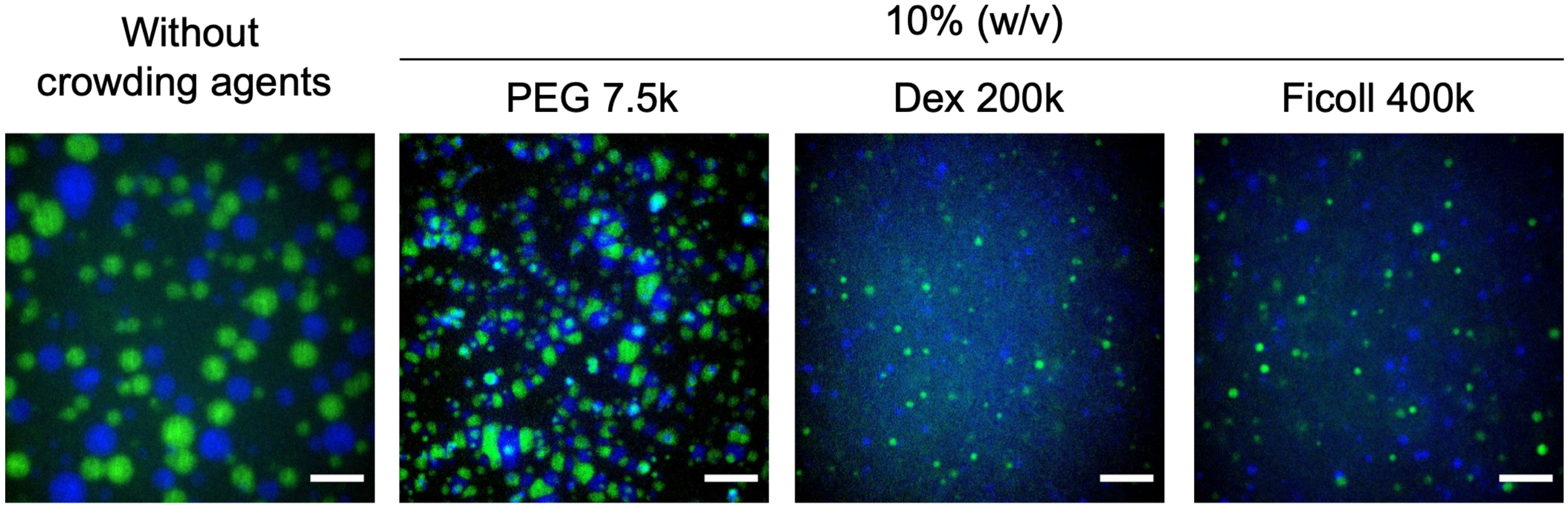

Figure S8. Effects of crowding agents on A/B-droplet contact-pattern formation. Fluorescence microscopy images of A- and B-droplets in the absence of a crowding agent or in the presence of 10% (w/v) PEG 7.5k, Dex 200k, or Ficoll 400k. The samples were prepared in PCR tubes according to the reagent concentrations listed in Table S10, annealed at 95 °C for 3 min, and then cooled to 25 °C at a rate of −1 °C/min. After incubation on a stage heater for 10 min at 60 °C in the absence of a crowding agent or at 65 °C in the presence of 10% (w/v) PEG 7.5k, Dex 200k, or Ficoll 400k, the samples were observed by fluorescence microscopy (Olympus). Scale bars: 20 μm.

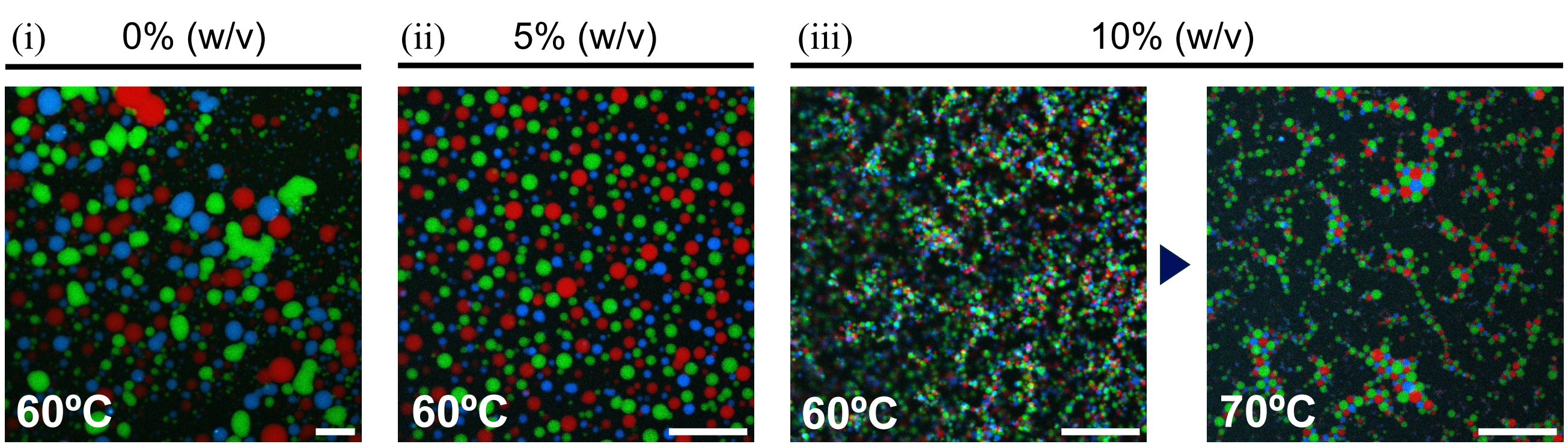

Figure S9. Wide-field fluorescence microscopy images of networked patterns formed by A-, B-, and C-droplets composed of NS-A, NS-B, and NS-C, respectively, at different PEG 7.5k concentrations. The confocal laser-scanning microscope (Nikon) was used for observation. Scale bars: 50 μm.

2. Supplementary Tables

Table S1. Oligonucleotide sequences of Y-shaped DNA nanostructures. The sticky end region is indicated in bold font. Complementary stem sequences are denoted by the same color.

Name	Sequence (5'→ 3')
Y_{A1}_8	**GCTCGAGC**CAGTGAGGACGGAAGTTTGTCGTAGCATCGCACC
Y_{A2}_8	**GCTCGAGC**CAACCACGCCTGTCCATTACTTCCGTCCTCACTG
Y_{A3}_8	**GCTCGAGC**GGTGCGATGCTACGACTTTGGACAGGCGTGGTTG
Y_{A1}_0	CAGTGAGGACGGAAGTTTGTCGTAGCATCGCACC
Y_{A2}_0	CAACCACGCCTGTCCATTACTTCCGTCCTCACTG
Y_{A3}_0	GGTGCGATGCTACGACTTTGGACAGGCGTGGTTG
Y_{A2}_0_5'-FAM	**[FAM]-**CAACCACGCCTGTCCATTACTTCCGTCCTCACTG
Y_{A3}_8_inter-FLU	**GCTCGAGC**GGTGCGATGC-**[FLU-**dT**]**-ACGACTTTGGACAGGCGTGGTTG
Y_{B1}_8	**CTCGCGAG**AAAGGAACTCTCCGCGTTGACAAAGCCGACACGT
Y_{B2}_8	**CTCGCGAG**GCCTCTGTGTCGCATCTTCGCGGAGAGTTCCTTT
Y_{B3}_8	**CTCGCGAG**ACGTGTCGGCTTTGTCTTGATGCGACACAGAGGC
Y_{B2}_0_5'-Alexa405	**[Alexa405]-**GCCTCTGTGTCGCATCTTCGCGGAGAGTTCCTTT
Y_{C1}_8	**CAGCGCTG**CTGGTTACACTGAGCTTTATGAACCTAGTGTGGC
Y_{C2}_8	**CAGCGCTG**GCCACACTAGGTTCATTTCGCTTGATACGATGTC
Y_{C3}_8	**CAGCGCTG**GACATCGTATCAAGCGTTAGCTCAGTGTAACCAG
Y_{C2}_0_5'-Cy5	**[Cy5]-**GCCACACTAGGTTCATTTCGCTTGATACGATGTC

Table S2. Concentrations of reagents used to examine the effect of crowding agents on the phase-separation temperature by microscopic observation ($T_{p,m}$).

Reagents	Final concentration
Y_{A1}_8	5 μM
Y_{A2}_8	4.5 μM
Y_{A3}_8	5 μM
Y_{A2}_0_5'-FAM	0.5 μM
PEG 7.5k, Dex 200k or Ficoll 400k	0 or 10% (w/v)
Tris-HCl (pH 7.5)	20 mM
NaCl	350 mM

Table S3. Concentrations of reagents employed to investigate the influence of PEG molecular weight on the phase-separation temperature through turbidity measurement ($T_{p,t}$). The symbol c^* represents the overlap concentration of polyethylene glycol (PEG) for each molecular weight. The overlap concentrations of PEG 0.6k, 1k, 3k, 7.5k, and 8k are 33.1, 22.6, 9.92, 4.99, and 4.76% (w/v), respectively. The overlap concentration is traditionally defined in terms of (w/v); thus, % (w/v) is used instead of % (w/w) in this study. Because the density of the PEG solution is ~1 g/cm³ at PEG concentrations of around 10%(w/w) range or lower, values in the '% (w/v)' unit are assumed to be the same as those in the '% (w/w)' unit.

**Reagents**	**Final concentration**
Y_{A1}_8	5 µM
Y_{A2}_8	5 µM
Y_{A3}_8	5 µM
PEG 0.6k, 1k, 3k, 7.5k, or 8k	0, 10, or c^* % (w/v)
Tris-HCl (pH 7.5)	20 mM
NaCl	350 mM

Table S4. Concentrations of reagents used to assess the impact of PEG 7.5k and NaCl concentration on the $T_{p,t.}$

**Reagents**	**Final concentration**
Y_{A1}_8	5 µM
Y_{A2}_8	5 µM
Y_{A3}_8	5 µM
PEG 7.5k	0, 5, or 10% (w/v)
Tris-HCl (pH 7.5)	20 mM
NaCl	200, 350, or 600 mM

Table S5. Concentrations of reagents utilized for the analysis of intensity ratios and the estimation of dilute-phase concentration.

**Reagents**	**Final concentration**
Y_{A1}_8	5 µM
Y_{A2}_8	5 µM
Y_{A3}_8	4.5 µM
Y_{A3}_8_inter-FLU	0.5 µM
PEG 7.5k	0 or 10% (w/v)
Tris-HCl (pH 7.5)	20 mM
NaCl	350 mM

Table S6. Concentrations of reagents used to prepare the calibration curve for estimating the dilute-phase concentration.

**Reagents**	**Final concentration**
Y_{A1}_0	5 µM
Y_{A2}_0	5 µM
Y_{A3}_0	4.5 µM
Y_{A3}_8_inter-FLU	0.5 µM
PEG 7.5k	0 or 10% (w/v)
Tris-HCl (pH 7.5)	20 mM
NaCl	350 mM

Table S7. Concentrations of reagents utilized for examining the self-organization pattern formation of two types of DNA droplets, influenced by PEG 7.5k and NaCl concentration.

**Reagents**	**Final concentration**	
	PEG 7.5k concentration–dependent experiments	NaCl concentration–dependent experiments
Y_{A1}_8	5 µM	5 µM
Y_{A2}_8	4.5 µM	4.5 µM
Y_{A3}_8	5 µM	5 µM
Y_{A2}_0_5'-FAM	0.5 µM	0.5 µM
Y_{B1}_8	5 µM	5 µM
Y_{B2}_8	4.5 µM	4.5 µM
Y_{B3}_8	5 µM	5 µM
Y_{B2}_0_5'-Alexa405	0.5 µM	0.5 µM
PEG 7.5k	0, 5, 10, or 20% (w/v)	10 % (w/v)
Tris-HCl (pH 7.5)	20 mM	20 mM
NaCl	350 mM	200, 350 or 600 mM

Table S8. Concentrations of reagents used to study the self-organization pattern formation of two distinct types of DNA droplets, influenced by the concentration ratio of NS-A and NS-B.

Reagents	Final concentration				
	$Y_A : Y_B = 8 : 2$	$Y_A : Y_B = 6.7 : 3.3$	$Y_A : Y_B = 5 : 5$	$Y_A : Y_B = 3.3 : 6.7$	$Y_A : Y_B = 2 : 8$
Y_{A1}_8	8 µM	6.7 µM	5 µM	3.3 µM	2 µM
Y_{A2}_8	7.2 µM	6.03 µM	4.5 µM	3 µM	1.8 µM
Y_{A3}_8	8 µM	6.7 µM	5 µM	3.3 µM	2 µM
Y_{A2}_0_5'-FAM	0.8 µM	0.67 µM	0.5 µM	0.33 µM	0.2 µM
Y_{B1}_8	2 µM	3.3 µM	5 µM	6.7 µM	8 µM
Y_{B2}_8	1.8 µM	3 µM	4.5 µM	6.03 µM	7.2 µM
Y_{B3}_8	2 µM	3.3 µM	5 µM	6.7 µM	8 µM
Y_{B2}_0_5'-Alexa405	0.2 µM	0.33 µM	0.5 µM	0.67 µM	0.8 µM
PEG 7.5k	10 % (w/v)	10 % (w/v)	10 % (w/v)	10 % (w/v)	10 % (w/v)
Tris-HCl (pH 7.5)	20 mM	20 mM	20 mM	20 mM	20 mM
NaCl	350 mM	350 mM	350 mM	350 mM	350 mM

Table S9. Concentrations of reagents used to study the self-organization pattern formation in three different types of DNA droplets.

Reagents	Final concentration
Y_{A1}_8	5 µM
Y_{A2}_8	4.5 µM
Y_{A3}_8	5 µM
Y_{A2}_0_5'-FAM	0.5 µM
Y_{B1}_8	5 µM
Y_{B2}_8	4.5 µM
Y_{B3}_8	5 µM
Y_{B2}_0_5'-Alexa405	0.5 µM
Y_{C1}_8	5 µM
Y_{C2}_8	4.5 µM
Y_{C3}_8	5 µM
Y_{C2}_0_5'-Cy5	0.5 µM
PEG 7.5k	0, 5, 7.5 or 10% (w/v)
Tris-HCl (pH 7.5)	20 mM
NaCl	350 mM

Table S10. Reagent concentrations for A- and B-droplet contact experiments with different crowding agents.

Reagents	Final concentration
Y_{A1}_8	5 μM
Y_{A2}_8	4.5 μM
Y_{A3}_8	5 μM
Y_{A2}_0_5'-FAM	0.5 μM
Y_{B1}_8	5 μM
Y_{B2}_8	4.5 μM
Y_{B3}_8	5 μM
Y_{B2}_0_5'-Alexa405	0.5 μM
PEG 7.5k, Dex 200k, or Ficoll 400k	0 or 10% (w/v)
Tris-HCl (pH 7.5)	20 mM
NaCl	350 mM

3. Supplementary Reference

(1) Devanand, K.; Selser, J. C. Asymptotic Behavior and Long-Range Interactions in Aqueous Solutions of Poly(Ethylene Oxide). *Macromolecules* **1991**, *24* (22), 5943–5947.
